# Two Years of Expanded Newborn Screening in Russia: High-Throughput Detection of Inherited Metabolic Disorders by Tandem Mass Spectrometry with Next-Generation Sequencing Confirmation

**DOI:** 10.3390/ijns12010013

**Published:** 2026-03-02

**Authors:** Ekaterina Y. Zakharova, Galina V. Baydakova, Polina V. Baranova, Darya Y. Aleksandrova, Olga A. Shchagina, Yulia S. Itkis, Natalya V. Milovanova, Tatyana S. Nagornova, Olga N. Ivanova, Yana D. Nazarenko, Sergey V. Voronin, Alena L. Chukhrova, Varvara A. Kadnikova, Ekaterina E. Lotnik, Nina V. Ryadninskaya, Aleksander V. Polyakov, Kirill V. Savostyanov, Fanil S. Bilalov, Alexander L. Koroteev, Dmitry Y. Trofimov, Tatyana A. Bairova, Gulnara N. Seitova, Sergei V. Mordanov, Svetlana A. Matulevich, Elena B. Nikolaeva, Sergey I. Kutsev

**Affiliations:** 1 Research Centre for Medical Genetics, 1 Moskvorechye St., 115522 Moscow, Russia; 2 National Medical Research Center for Children’s Health, 2 b.1 Lomonosovsky Ave., 119991 Moscow, Russia; 3 Republican Medical Genetic Centre, 74 Gafuri St., 450076 Ufa, Russia; 4 Diagnostic Centre (Medical Genetic), 5A Tobolskaya St., 194044 Saint-Petersburg, Russia; 5 National Medical Research Center for Obstetrics, Gynecology and Perinatology Named After Academician V.I. Kulakov, 4 Akademik Oparin St., 117997 Moscow, Russia; 6 Scientific Centre for Family Health and Human Reproduction Problems, 16 Timiryazev St., 664003 Irkutsk, Russia; 7 Tomsk National Research Medical Centre of the Russian Academy of Sciences, 10 Naberezhnaya Reki Ushaiki St., 634050 Tomsk, Russia; 8 Laboratory Department of the Medical Genetic Center, Rostov State Medical University, 29 Nakhichevanskiy Lane, 344022 Rostov-on-Don, Russia; 9 Regional Clinical Hospital No 1 Named After Professor S.V. Ochapovsky, 167 May 1 St., 350086 Krasnodar, Russia; 10 Mother and Child Healthcare Clinical Diagnostic Centre, 52 Flotskaya St., 620067 Yekaterinburg, Russia

**Keywords:** expansion of newborn screening, inherited metabolic diseases, Russian Federation

## Abstract

In 2023, the Russian Federation expanded its national newborn screening (NBS) program from 5 to 36 conditions, 29 of which are inherited metabolic diseases (IMDs). This study presents the first nationwide results and outcomes of the expanded NBS program. Between January 2023 and December 2024, dried blood spots from 2,466,615 newborns (98.53% of the birth cohort) were analyzed for IMDs using MS/MS. Screen-positive cases were referred to the national reference center for confirmatory testing, which included biochemical (MS/MS and GC-MS) and genetic analyses (NGS). A total of 41,728 neonates (1.69%) screened positive, of whom 37,733 underwent confirmatory testing. It resulted in 834 confirmed diagnoses of IMDs (1 in 2900 live births). Phenylketonuria was the most prevalent IMD (*n* = 538; 1 in 4600), followed by MCADD (*n* = 99; 1 in 25,000). Distinct regional and ethnic variations were observed, including a high prevalence of tyrosinemia type 1 in the Chechen Republic and MCADD in North Ossetia. The integration of NGS was essential for resolving complex cases, such as identifying heterozygous carriers and dual diagnoses. These findings underscore the program’s clinical utility, highlight unique epidemiological patterns, and identify challenges such as false positives and diagnostic complexities, which will guide future refinements.

## 1. Introduction

Inherited metabolic disorders (IMDs) are a group of rare and often severe diseases that typically manifest in infancy. Early detection through newborn screening (NBS) facilitates prompt diagnosis, enabling patients to receive pre-symptomatic treatment and thereby improving clinical outcomes [1]. NBS programs vary globally in the number of diseases screened, confirmatory diagnostic algorithms, timing of sample collection, and overall organization of the screening process [2,3].

NBS for phenylketonuria (PKU), one of the most common IMDs, was first implemented in the 1960s. Since then, the panel of screened disorders has expanded significantly, driven by advances in laboratory technologies—most notably tandem mass spectrometry (MS/MS). According to the American College of Medical Genetics (ACMG) criteria, 29 conditions detectable by MS/MS are considered appropriate for NBS [4].

The potential of next-generation sequencing (NGS) as a first-tier NBS test has been widely advocated. However, its application remains limited to pilot projects, with preliminary results anticipated in several countries [5,6,7]. Meanwhile, NGS—either alone or in combination with biochemical assays—has been successfully implemented in numerous screening programs as an effective confirmatory diagnostic strategy [5,6,7].

In 1993, the Russian Federation initiated screening for PKU and congenital hypothyroidism. In 2006, the NBS program was expanded to include congenital adrenal hyperplasia, galactosemia, and cystic fibrosis. Starting from 1 January 2023, the NBS program further expanded from the standard five conditions to thirty-six inherited and/or congenital diseases, 29 of which are IMDs.

The purpose of this paper is to present the results of the expanded NBS program in the Russian Federation during 2023–2024, which incorporated MS/MS testing and confirmatory NGS diagnostics. This study specifically focuses on IMDs detected by NBS using MS/MS. Data on screening outcomes for biotinidase deficiency, spinal muscular atrophy (SMA), and primary immunodeficiencies (PIDs)—which are also included in the expanded NBS program—are not addressed in this article.

## 2. Materials and Methods

### 2.1. NBS Program Infrastructure

Participation in the NBS program is voluntary and based on informed consent, although it is not documented in writing. Parents have the option to decline participation in the NBS program by submitting a handwritten statement of voluntary renunciation with their signatures if the family is not willing to participate (Opt-out consent) in accordance with Federal Law No. 323-FZ. NBS program is a nationwide public health program regulated by orders of the Ministry of Health (including Order No. 274n).

Blood sampling for dried blood spots (DBS) is conducted at the maternity hospital, perinatal center, or the hospital where the newborn is receiving care. Filter papers are sent to the respective interregional medical centers for further screening within 48 h of sample collection. First-tier diagnostic tests include MS/MS-based screening for IMDs within 72 h after birth. The primary goal of the first stage of screening is to conduct mass NBS to identify at-risk groups.

**Figure 1:**
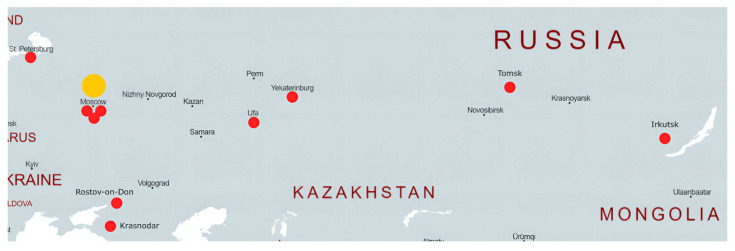
Regional organization of NBS in the Russian Federation. The big yellow dot indicates the national reference centre (Research Centre for Medical Genetics in Moscow), and the small red dots indicate ten interregional NBS centers: (1) National Medical Research Center for Obstetrics, Gynecology and Perinatology named after academician V.I. Kulakov (Moscow); (2) National Medical Research Center for Children’s Health (Moscow); (3) Morozov Children’s City Clinical Hospital of the Moscow City Healthcare Department (Moscow); (4) Tomsk National Research Medical Center of the Russian Academy of Sciences (Tomsk); (5) Scientific Centre for Family Health and Human Reproduction Problems (Irkutsk); (6) Rostov State Medical University (Rostov-on-Don); (7) Diagnostic Centre (Medical Genetic) (Saint Petersburg); (8) Regional Clinical Hospital No 1 named after Professor S.V. Ochapovsky (Krasnodar); (9) Republican Medical Genetics Centre (Ufa); and (10) Mother and Child Healthcare Clinical Diagnostic Centre (Yekaterinburg). Base map generated with MapChart.net.

**Figure 2:**
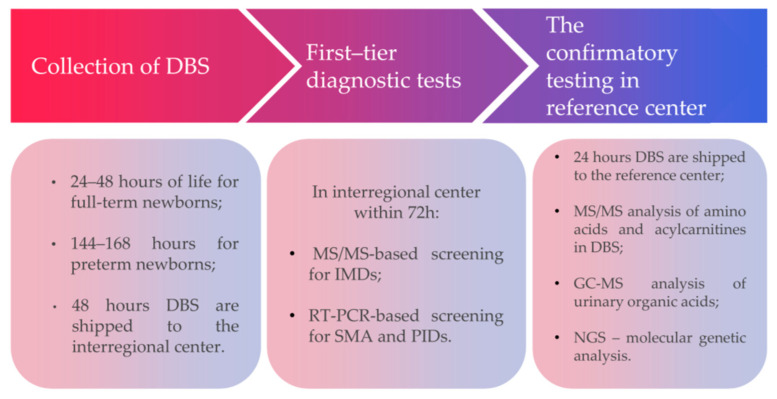
Algorithm of newborn screening in the Russian Federation. DBS = dried blood spots; IMDs = inherited metabolic disorders; GC-MS = gas chromatography-mass spectrometry; PIDs = primary immunodeficiencies; SMA = spinal muscular atrophy; MS/MS = tandem mass spectrometry; RT-PCR = Real-time PCR.

All subjects (regions) of the Russian Federation are assigned to 10 NBS centers that perform the quantification of amino acids and acylcarnitines using MS/MS (Figure 1). All positive or inconclusive NBS results undergo confirmatory biochemical and genetic testing at the national reference center, the Research Centre for Medical Genetics in Moscow. Confirmatory testing includes MS/MS-based analysis of amino acids and acylcarnitines, gas chromatography–mass spectrometry (GC-MS) analysis of urinary organic acids, and NGS-based molecular genetic analysis (Figure 2). Clinical geneticists employed at the interregional NBS units evaluate the results and report positive screening results to patients and/or pediatricians at the infant’s local hospital within 24 h of obtaining a positive first-round result. If necessary, a specialist from the respective region of the Russian Federation promptly refers a newborn from a high-risk group for hospitalization and prescribes specialized dietary or other pathogenetic therapies until confirmatory screening results are available. Once the diagnosis is confirmed, therapy is either continued if previously prescribed or initiated based on the identified disease. Further follow-up care is conducted in accordance with established clinical guidelines.

### 2.2. Primary NBS Diagnostics

#### 2.2.1. DBS Collection

The recommended time for heel blood collection on two Whatman 903 filter papers (DBS) is 24–48 h after birth (not earlier than 3 h after feeding) for full-term newborns, and 144–168 h after birth for preterm newborns. If a neonate undergoes transfusion therapy or extracorporeal membrane oxygenation, the DBS should be collected either before these procedures or no earlier than 48–72 h afterward. For neonates in the intensive care unit at the time of blood collection, it is recommended to retest the blood on the 14th day of life or before discharge, whichever occurs first. Capillary blood samples are collected on filter cards, dried, and sent by priority mail to one of the ten NBS centers.

#### 2.2.2. MS/MS-Based Analysis of Amino Acids and Acylcarnitines in DBS

Quantification of amino acids and acylcarnitines in DBS is conducted using MS/MS QSight 225MD UHPLC Screening System (Waltham, MA 02451, USA); NeoBase 2 NBS kit (PerkinElmer, Turku, Finland). Cutoff values for amino acids and acylcarnitines, elevations of which may indicate an IMD included in the expanded NBS, are provided in the Supplementary Materials (Table S1). Concentrations of amino acids and acylcarnitines are expressed in µmol/L.

### 2.3. Confirmatory Diagnostics

#### 2.3.1. Urine Sample Collection

For the analysis of urinary organic acids using GC-MS, a morning urine sample is collected in a sterile container. The samples are transported on dry ice and then stored at −20 °C.

#### 2.3.2. Biochemical Tests

Confirmatory biochemical tests include quantifying amino acids and acylcarnitines in DBS using MS/MS. Additionally, when blood test results indicate organic aciduria, urinary organic acids are analyzed by GC-MS.

##### MS/MS-Based Analysis of Amino Acids and Acylcarnitines in DBS

Similarly, the QSight 225MD UHPLC Screening System (Waltham, MA 02451, USA) is used to analyze amino acid and acylcarnitine profiles with the NeoBase 2 NBS kit (PerkinElmer, Turku, Finland). Cutoff values for amino acids and acylcarnitines—elevations of which may indicate an inherited disorder included in the expanded NBS—are provided in the Supplementary Materials (Table S1). Cutoff values were established based on the 99.5th percentiles. Concentrations of amino acids and acylcarnitines are expressed in µmol/L.

##### GC-MS Analysis of Urinary Organic Acids

As part of the confirmatory diagnostics, if urine samples are available, organic acids are measured using GC-MS (GCMS-TQ8050 EI, SHIMADZU) (1, Nishinokyo-Kuwabara-cho, Nakaguo-ku, Kyoto, 604, Japan). Sample preparation involves extraction with organic solvents at a pH of 1–2, followed by derivatization using methoxyamine hydrochloride and N,O-Bis(trimethylsilyl)trifluoroacetamide.

GC-MS is performed on newborns exhibiting primary alterations indicative of disorders associated with elevated levels of specific organic acids in the urine, such as propionic acidemia, methylmalonic aciduria, and biotinidase deficiency.

Cutoff values for organic acids, elevations of which may indicate an inherited disorder included in the expanded NBS program, are provided in the Supplementary Materials (Table S2). The concentration of organic acids is expressed in mmol/mol of creatinine. Creatinine concentration in urine is determined using the Jaffe method, which is based on the reaction of creatinine with picric acid under alkaline conditions.

#### 2.3.3. Molecular Genetic Analysis

Genomic DNA is isolated from DBS using the MagPure Forensic DNA Kit (Magen Biotechnology Co., Guangzhou, China) according to the manufacturer’s standard protocol. DNA concentration is measured with a Qubit 2.0 fluorometer using Qubit BR and Qubit HS reagents (Thermo Fisher Scientific, Waltham, MA, USA) following the manufacturer’s instructions. Additionally, genomic DNA is extracted from whole blood using the Wizard^®^ Genomic DNA Purification Kit (Promega, Madison, WI, USA) according to the manufacturer’s protocol.

For newborns in the PKU risk group, frequent pathogenic variants in the *PAH* gene (NM_000277) are detected using a custom system based on an allele-specific ligase reaction. The targeted variants include: c.47_48del (p.Ser16*), c.143T>C (p.Leu48Ser), c.168+5G>A (IVS2+5G>A), c.168+5G>C (IVS2+5G>C), c.331C>T (p.Arg111*), c.441+5G>T (IVS4+5G>T), c.442-2913_509+1173del4154ins268 (EX5del4154ins268), c.473G>A (p.Arg158Gln), c.664_665del (p.Asp222*), c.727C>T (p.Arg243*), c.728G>A (p.Arg243Gln), c.754C>T (p.Arg252Trp), c.781C>T (p.Arg261*), c.782G>A (p.Arg261Gln), c.838G>A (p.Glu280Lys), c.842C>T (p.Pro281Leu), c.898G>T (p.Ala300Ser), c.916A>G (p.Ile306Val), c.1045T>C (p.Ser349Pro), c.1066-11G>A (IVS10-11G>A), c.1169A>G (p.Glu390Gly), c.1208C>T (p.Ala403Val), c.1222C>T (p.Arg408Trp), c.1241A>G (p.Tyr414Cys), and c.1315+1G>A (IVS12+1G>A). Following polyacrylamide gel electrophoresis and ethidium bromide staining, the products are visualized under UV light.

A targeted custom NGS panel was developed to analyze genes associated with other IMDs included in the MS/MS-based screening. The gene panel covers the coding sequences and adjacent intronic regions of 92 genes, including those associated with conditions not currently included in the NBS program in the Russian Federation but potentially detectable through biochemical testing (Supplementary Materials (Table S3).

In cases where NGS analysis identifies only a single heterozygous variant in a gene, but the biochemical profile clearly indicates the presence of a disease, whole-genome sequencing (WGS) is performed as part of a research study to further investigate the genetic basis of the condition.

NGS is performed using a MiSeq system Illumina (5200 Illumina Way, San Diego, CA 92122, USA), achieving an average coverage depth of at least 200×, with targeted regions covered at a minimum of 60× in 95–98% of cases. The initial analysis of the sequencing data was conducted using the standard automated algorithm provided by Illumina.

Identified variants were annotated using the nomenclature provided on the website http://varnomen.hgvs.org/recommendations/DNA (accessed on 23 February 2026; version 21.1.0). Population frequencies of the identified variants were assessed using data from the 1000 Genomes Project, ESP6500, and the Genome Aggregation Database (gnomAD) v4.1.0. The clinical significance of the variants was evaluated using the OMIM database and the HGMD professional pathogenic variants database (version 2021.3). The pathogenicity and causality of the genetic variants were assessed in accordance with international guidelines (ACMG/AMP) for interpreting data obtained through massive parallel sequencing.

To determine the copy number of exons in the *PAH* gene, the SALSA^®^ MLPA^®^ Probemix P055 PAH kit (MRC Holland, Amsterdam, The Netherlands) was used according to the manufacturer’s protocol, followed by fragment analysis on the ABI Prism^®^ 3500 Genetic Analyzer (Thermo Fisher Scientific, Waltham, MA, USA).

## 3. Results

A total of 2,466,615 neonates were born in the Russian Federation between January 2023 and December 2024 (Table 1). Of these, 1,231,401 blood samples (98.03% coverage in 2023) and 1,199,015 blood samples (99.06% coverage in 2024) were delivered to ten designated NBS centers for primary screening in 2023 and 2024, respectively. The total number of samples delivered was 2,430,416. Coverage of the expanded NBS program increased from 98.03% in 2023 to 99.06% in 2024. Out of the 2,425,368 samples that underwent primary screening (98.32% of the delivered samples), 41,728 were identified as at risk for hereditary diseases. Among these, 30,103 samples (72.14%) were sent to the Research Centre for Medical Genetics for confirmatory diagnosis: 16,335 (71.71%) in 2023 and 13,768 (72.65%) in 2024 (Table 1).

### 3.1. Inherited Metabolic Diseases (IMDs)

**Figure 3:**
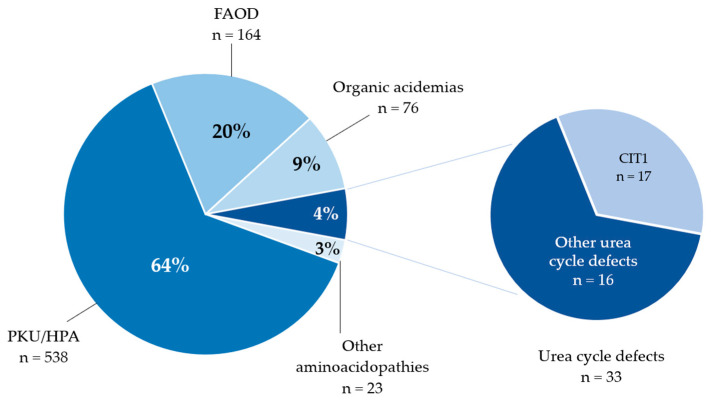
Distribution of IMDs diagnosed during the expanded NBS program in the Russian neonatal population (2023–2024). IMDs = inherited metabolic diseases; FAOD = fatty acid β-oxidation disorders; CIT1 = citrullinemia type 1; HPA = hyperphenylalaninemia; PKU = phenylketonuria.

In 2023–2024, a total of 834 confirmed cases of IMDs were identified through NBS in the Russian Federation (Figure 3, Table 2) with fully confirmed genotypes in a homozygous or compound heterozygous state. Subsequent calculations of disease frequency were based exclusively on this patient group. Pseudodeficiency was ruled out based on the results of biochemical tests and the patient’s clinical presentation. In cases where biochemical findings were inconsistent with the results of NGS analysis and/or clinical symptoms were absent or did not align with the suspected diagnosis, the family was referred for genetic counseling to determine further examination tactics.

The median age at diagnosis was 32 days (range: 10–419 days). The overall incidence rate of IMDs detected by MS/MS was 1 in 2900 (95% CI: 1:2800–1:3150) live births (1 in 3300 in 2023, and 1 in 2700 in 2024).

Among 30,103 samples referred for confirmatory testing, heterozygous variants were detected in 190 cases (0.63%). These patients were referred for further investigation using whole-genome sequencing (WES) to identify a second pathogenic variant in the relevant gene or to confirm true heterozygous carrier status.

#### 3.1.1. Aminoacidopathies

Aminoacidopathies represented the most prevalent category of IMDs, with 559 confirmed cases, accounting for 67% of all IMDs.

Phenylketonuria/hyperphenylalaninemia (PKU/HPA) was the most frequently diagnosed IMDs, accounting for 538 cases (64.51% of all IMDs), with an incidence of 1 in 4600 live births.

PKU had the highest number of patients in the Southern and North Caucasus regions compared to other regions of the Russian Federation. These regions reported 86 patients (15.98%, or 1 in 3200) and 79 patients (14.68%, or 1 in 3200), respectively (*n* = 165; 30.66% of all PKU cases).

Between 2023 and 2024, the highest number of patients diagnosed with tyrosinemia type 1 (TYR I, *n* = 13) was reported in the Chechen Republic (*n* = 4; 1 in 14,700) and the Republic of Buryatia (*n* = 3; 1 in 6700). One of the most common *FAH* variants in the Russian Federation is c.1025C>T (p.Pro342Leu), which is frequently observed in the Chechen population. The frequency of TYR I is 1 in 16,020, making it one of the highest rates worldwide [8]. Additionally, three patients from the Republic of Buryatia were found to be homozygous for the c.1090G>C (p.Glu364Gln) variant. The common European *FAH* variants (c.1062+5G>A and c.554-1G>T) were rare in the Russian Federation (7.7% and 3.9%) [9].

#### 3.1.2. Urea Cycle Disorders

Among the urea cycle disorders (UCDs), which account for 3.95% of all IMD cases (*n* = 33), the most prevalent conditions were citrullinemia type 1 (CIT1; 1 in 145,000; 95% CI: 1 in 98,000 to 1 in 228,000) and argininosuccinic aciduria (ASA; 1 in 177,000; 95% CI: 1 in 114,000 to 1 in 292,000).

#### 3.1.3. Fatty Acid Oxidation Disorders

Fatty acid oxidation disorders (FAODs) accounted for 19.66% of all IMD cases (*n* = 164). Medium-chain acyl-CoA dehydrogenase deficiency (MCADD; 1 in 25,000 live births; 95% CI: 1 in 21,200–1 in 29,700 live births) and very long-chain acyl-CoA dehydrogenase deficiency (VLCADD; 1 in 110,500 live births; 95% CI: 1 in 72,100–1 in 176,000 live births) were the most prevalent FAODs, representing 99 (60.36%) and 22 (13.41%) cases, respectively. The highest number of neonates diagnosed with MCADD was identified in North Ossetia, with 15 patients (15.5%, or 1 in 1050 live births).

Among FAODs, long-chain 3-hydroxyacyl-CoA dehydrogenase deficiency (LCHADD) was the third most prevalent condition, with 17 confirmed cases (10.36%, or 1 in 145,000 live births; 95% CI: 1 in 98,000 to 1 in 216,000 live births).

#### 3.1.4. Organic Acidemias

Additionally, 76 neonates (9.11% of all IMDs) were diagnosed with organic acidemias: 26 neonates (34.21%) with glutaric acidemia type 1 (GAI), 17 neonates (22.36%) with isovaleric acidemia (IVA), 26 neonates (34.21%) with various types of methylmalonic aciduria (MMA), and 2 neonates (2.63%) with propionic aciduria (PA).

### 3.2. IMD-Associated Variants Identified During NBS

NBS identified 355 variant alleles associated with 29 IMDs, of which 114 (32.11%) were novel variants not previously reported in the HGMD, thereby expanding the genetic spectrum of IMDs.

The most prevalent variants were identified in five genes: *PAH*, *ACADM*, *HADHA*, *GCDH*, and *FAH* (Table 3).

### 3.3. Disorders and Conditions Beyond the Expanded NBS Program

While our targeted sequencing panel primarily focuses on conditions included in the NBS program, it also incorporates genes associated with clinically overlapping disorders and conditions to facilitate differential diagnosis. Thus, a total of 24 such cases (2.87% of all IMDs confirmed at the Research Centre for Medical Genetics) were identified.

The majority of these cases involved patients with 3-methylcrotonyl-CoA carboxylase deficiency, totaling 19 cases (7 cases of 3-methylcrotonyl-CoA carboxylase 1 deficiency, *MCCC1*, and 12 cases of 3-methylcrotonyl-CoA carboxylase 2 deficiency, *MCCC2*). According to the literature [10,11], this is one of the most common causes of elevated C5OH levels detected during NBS. Biotinidase deficiency rarely causes an increase in this biomarker during the neonatal period, and other conditions are extremely rare.

This study also identified two rare cases of methylmalonic aciduria (MMA) in the Russian Federation: one patient (allele frequency 4.16%) with MMA and homocysteinemia, cblX type (*HCFC1*), and another patient (allele frequency 4.16%) with MMA and homocystinuria, cblF type (*LMBRD1*). Additionally, three patients with ornithine transcarbamylase deficiency (*OTC*) were identified, including one case with a large deletion spanning chrX:38,331,343–38,348,676.

### 3.4. Unusual Findings: Dual Diagnoses and Complex Genotypes

The implementation of multi-gene sequencing panels in NBS has revealed a higher-than-expected prevalence of complex genetic scenarios. These include dual molecular diagnoses (the co-occurrence of two distinct IMDs), the presence of an IMD alongside pathogenic variants in one or more other disease-associated genes, and carrier states. Over the course of two years of NBS, two such cases were identified (Table 4).

Patient 1, from the Republic of North Ossetia-Alania (North Caucasus Federal District), is a full-term male born at 39 weeks with a birth weight of 3350 g. No parental data is available. Primary screening revealed elevated levels of C6, C8, C10, C10:1, and Phe. MS/MS analysis confirmed elevated Phe (138.774 μmol/L) and C8 levels (0.796 μmol/L). Molecular analysis identified a homozygous variant in the *ACADM* gene, which is the second most frequent variant in the Russian Federation. Concurrently, testing of the PAH gene revealed pathogenic variants in a compound heterozygous state.

Patient 2, from Sverdlovsk Oblast (Ural Federal District), is a full-term male weighing 3580 g, with no available parental data. He has two siblings, aged 2 and 5, both born in Tajikistan. Primary screening revealed decreased C0 levels (5.849 μmol/L), which were confirmed by MS/MS analysis in DBS (6.614 μmol/L) and plasma (5.325 μmol/L). Genetic testing identified homozygous variants in the *SLC22A5* and *ETFB* genes.

## 4. Discussion

In 2023–2024, a total of 834 confirmed cases of IMDs were identified through expanded NBS in the Russian Federation. These data are comparable to those reported in Europe and Asia. For example, the incidence of IMDs is 1 in 4900 in Denmark and 1 in 2517 in Germany [4]. In Korea, the incidence is 1 in 2000; in Japan, 1 in 9330; and in Hong Kong, 1 in 4122 [12]. The highest frequency of IMDs is observed in Qatar, with a rate of 1 in 1327, which may be attributed to the high prevalence of consanguineous marriages [13]. Similarly, the percentage of confirmed diagnoses among all detected NBS cases in the Southern and North Caucasian Federal Districts—where intra-national marriages are more common—was 1 in 2200 (95% CI: 1:2000–1:2500).

The most prevalent variants were identified in the following five genes: *PAH*, *ACADM*, *HADHA*, *GCDH*, and *FAH*.

Phenylketonuria/hyperphenylalaninemia (PKU/HPA) was the most frequently diagnosed IMD, accounting for 538 cases (64.04% of all IMDs), with an incidence of 1 in 4600 live births. This frequency aligns with reports from several European countries [14,15]. For example, the incidence of PKU has been reported as 1 in 4000 in Italy, 1 in 3000 in Sweden, and 1 in 143,000 in Japan [16]. The most frequent *PAH* variant in the Russian Federation was c.1222C>T (p.Arg408Trp) (46.06%), consistent with its high prevalence in Europe [17]. The second most common variant was c.1208C>T (p.Ala403Val) (6.79%), which is associated with Mediterranean populations [18].

FAODs accounted for 18.18% of all IMD cases (*n* = 152), with MCADD being the most prevalent FAOD in the Russian Federation. The observed incidence of MCADD was 1 in 25,000 births (95% CI: 1 in 21,200–1 in 29,700), comparable to rates reported in the Czech Republic [19] and Norway (1 in 27,139) [20]. However, these rates were significantly lower than those reported in Denmark (1 in 9164), the Netherlands (1 in 9624), Germany (1 in 4900), and Switzerland (1 in 11,500) [21].

The highest number of newborns with a confirmed diagnosis of MCADD in 2023–2024 was recorded in North Ossetia, with 15 cases (15.15%; 1 in 1050; 95% CI: 1:663–1:1540), accounting for 57.96% of all confirmed diagnoses in the region. The common variant NM_000016.5:c.388-19T>A (p.?) was identified in 13 patients from North Ossetia, with an allele frequency of 10.10% (20 alleles). In Denmark, this variant was detected in two newborns in a compound heterozygous state (c.244_245dup/c.388-19T>A) and was first identified through NBS [22].

For *HADHA*-related LCHADD, the third most prevalent FAOD, the homozygous incidence of the c.1528G>C variant was 1 in 205,000, which is similar to the incidence reported in Poland [23].

Organic acidemias accounted for 10.41% of all IMDs. Twenty-six neonates (29.89% of all organic acidemias) were diagnosed with various types of MMA. The overall incidence of MMA in the Russian Federation was 1 in 95,000 births (95% CI: 1 in 69,000 to 1 in 131,000). Global MMA frequencies exhibit considerable variability, ranging from 1 in 16,883 in China to 1 in 107,000 in Taiwan [24]. Our findings closely align with European reports: 1 in 56,000 in Spain and 1 in 85,000 in Portugal [3,25]. Notably, many neonatal screening studies do not specify the particular MMA subtypes detected. In our cohort, MMA-MUT (*n* = 19; 1 in 130,000; 95% CI:1 in 89,000 to 1 in 189,000) was the most prevalent subtype. This contrasts with reports from Italy and China, where MMA-CblC predominated, with significantly higher frequencies of 1 in 32,271 and 1 in 19,697, respectively [26].

The incidence of GA I in the Russian Federation was 1 in 95,000 live births (95% CI: 1 in 69,000 to 1 in 130,000), which is comparable to the estimated worldwide incidence of 1 in 100,000 live births in Western Europe [27]. In Japan, the incidence is estimated at 1 in 210,000 live births [28]. The incidence detected through NBS in Portugal is 1 in 160,000 [29]. Among 10,048 infants tested in Slovenia and 517,484 infants tested in China, no homozygotes were detected [30].

Among UCDs, which account for 3.9% of all IMD cases, the most prevalent conditions were CIT1 (1 in 145,000; 95% CI: 1 in 98,000 to 1 in 228,000) and ASA (1 in 177,000; 95% CI: 1 in 114,000 to 1 in 292,000). By comparison, the global incidence of CIT1 is estimated at 1 in 250,000 [31], while in Taiwan, it is reported as 1 in 188,380. The frequency of ASA in the Russian Federation is similar to that in Italy, where it is 1 in 189,740, with the highest frequency observed in Saudi Arabia at 1 in 16,847 [32].

Compared to the results of selective screening (SelS) conducted from 2021 to 2022, the expanded NBS program demonstrated an increased overall detection rate of IMDs (Table 5). The SelS targets a symptomatic population across all age groups. Individuals are included based on clinical suspicion of an IMD.

This increased detection rate of IMDs was most pronounced for disorders that often present with acute metabolic crises, such as VLCADD (0 SelS patients versus 22 NBS patients) and MCADD (3 SelS patients versus 99 NBS cases). Interestingly, the allele frequency of the common severe variant c.985A>G in the *ACADM* gene, associated with MCADD, decreased from 64.3% (SelS) to 38.4% (NBS). Similarly, the frequency of the severe homozygous c.1204C>T variant in the *GCDH* gene, associated with GA I, decreased from 67.4% (SelS) to 40.4% (NBS). This shift indicates that symptom-based screening primarily identifies a subset of patients with more severe, clinically manifest genotypes, whereas NBS reveals the full, often milder, genotypic and phenotypic spectrum of these diseases. These findings are consistent with reports from other countries [33,34,35,36].

The interpretation of heterozygous variants in screening was complicated by the fact that carriers of single pathogenic alleles in certain genes (e.g., *ACADM*, *ACADVL*, *SLC22A5*) may exhibit transient biochemical abnormalities during initial testing that normalize upon confirmatory analysis, mimicking patterns observed in affected patients. We observed cases in which patients with confirmed biallelic pathogenic variants also showed normalization of biochemical markers over time (e.g., *ACADM*, *ACADVL*). This phenotypic overlap between carriers and true compound heterozygotes underscores the necessity of comprehensive genetic confirmation and biochemical follow-up testing. Detecting heterozygous variants poses diagnostic challenges because some cases involve true carriers, while others conceal a second mutation identifiable only through advanced sequencing technologies.

### Limitations

The inclusion of 28 additional IMDs without prior pilot studies or established experience in interpreting borderline screening values inevitably introduces methodological challenges, particularly for certain disorders. Consequently, this has resulted in a high rate of false-positive results. The observed positive predictive value (PPV) of 3.87% indicates that, within our screening algorithm, only approximately 39 out of every 1000 screen-positive neonates ultimately received confirmed IMD diagnoses (Table 6). PPVs for certain IMDs are presented in the Supplementary Material (Table S4). It is noteworthy that while the overall PPV was low, for certain IMDs it reached 100%, such as for LCHADD.

DNA sequencing-based diagnostic methods frequently identify variants of uncertain significance (VUS) in at least one allele, leading to diagnostic uncertainty. In these cases, a definitive diagnosis can only be established through functional testing—such as enzymatic activity assays using patient dermal fibroblasts—which is often unavailable or challenging to perform, potentially causing significant delays in treatment initiation. However, such functional testing is performed on the patient through scientific programs. Despite risks that should be considered when adding genetic testing to the NBS program, NGS analysis significantly improves the accuracy of biochemical data interpretation.

Unfortunately, confirming a diagnosis often requires one to three months due to logistical challenges associated with sample collection, and analysis with confirmatory biochemical testing is completed within approximately 7 days, allowing early confirmation at the biochemical level. Following a positive screening result, parental segregation testing remains essential to confirm variant phasing (cis/trans configurations) and to exclude technical artifacts such as allele dropout. Existing technical limitations further complicate diagnosis, particularly in detecting copy number variants (CNVs), deep intronic variants, and other rare genomic alterations. Additionally, the Russian Federation currently lacks a nationwide metabolic patient registry. The absence of systematic data collection from the pre-expanded NBS era hinders direct comparisons of outcomes between cohorts identified before and after the implementation of expanded NBS.

## 5. Conclusions

In 2023, the NBS in the Russian Federation expanded from the standard five disorders to thirty-six inherited and/or congenital disorders, 29 of which are IMDs. This expansion has provided a comprehensive national overview and enhanced the capacity for diagnosing and managing metabolic disorders, along with secondary educational benefits and increased awareness among healthcare professionals.

Moving forward, the real-time evaluation of screening data using bioinformatics tools is essential for improving NBS programs. However, a significant challenge remains in detecting and managing attenuated phenotypes, such as VLCADD and MCADD, which place burdens on families and healthcare systems. The program also faced challenges, including a high false-positive rate with PPV of 3.87% and logistical delays in confirmatory testing.

The implementation of NBS using MS/MS has significantly expanded opportunities for early diagnosis. Between 2023 and 2024, screening of 2,466,615 newborns (achieving 98.33% coverage) detected IMDs, with an overall incidence of 1 in 2900. Compared to the previous period when patients were diagnosed only at the clinical stage, this approach has enabled the identification of milder disease forms. Additionally, certain ethnicity-specific patterns have been identified and confirmed, such as a high incidence of TYR I in the Chechen Republic and MCADD in North Ossetia. The application of second-tier tests has reduced the number of false-positive results, while the combined use of biochemical and DNA testing has provided deeper insights into biochemical variations in heterozygous carriers and age-related changes in biochemical parameters in patients diagnosed with conditions such as VLCADD and MCADD.

Early (preclinical) diagnosis of congenital and/or hereditary diseases through NBS ensures timely treatment, reduces infant mortality, and helps prevent lifelong disabilities and severe clinical complications. The program also supports family planning for mutation carriers. This is especially important in the Southern and North Caucasian Federal Districts, where consanguineous marriages are common and the prevalence of conditions such as MCADD and PKU is significantly higher than in other regions.

The identification of heterozygous carriers represents only the initial stage of diagnosis. All screen-positive cases subsequently undergo whole-genome sequencing to ensure comprehensive diagnostic resolution within the screening program.

## Figures and Tables

**Table 1:** Expanded Newborn Screening Results and Hereditary Disease Risk (2023–2024).

Year	A Total of Neonates Born	A Total of Samples Delivered	A Total of Samples That Underwent Primary Screening	Neonates at Risk for Hereditary Diseases	Samples Delivered for Confirmatory Diagnosis
2023	1,256,187 (100%)	1,231,401 (98.03% of total neonates)	1,227,130 (99.65% of samples delivered)	22,779 (1.86% of samples that underwent primary screening)	16,335 (71.71% of neonates at risk for hereditary diseases)
2024	1,210,428 (100%)	1,199,015 (99.06% of total neonates)	1,198,238 (99.93% of samples delivered)	18,949 (1.58% of samples that underwent primary screening)	13,768 (72.65% of neonates at risk for hereditary diseases)
Total	2,466,615 (100%)	2,430,416 (98.53% of total neonates)	2,425,368 (98.32% of samples delivered)	41,728 (1.69% of samples that underwent primary screening)	30,103 (72.14% of neonates at risk for hereditary diseases)

**Table 2:** The number of confirmed cases and incidence of hereditary disorders established during the expanded NBS in the Russian Federation in 2023–2024 (2,466,615 screened newborns).

Group	Disorder	Number of Confirmed Cases in 2023–2024	Incidence Estimated (95% CI)
Aminoacidopathies	PKU/HPA	538	1:4484 (1:4300–1:4900)
	HCU	4	1:617,000 (1:277,000–1:1,806,000)
	MSUD	4	Total 1:617,000 (1:277,000–1:1,806,000)
			BCKDHA -
			BCKDHB 1:617,000 (1:277,000–1:1,806,000)
			DBT -
	TYR I	13	1:190,000 (1:121,000–1:321,000)
FAOD	CPT I	2	1:1,234,000 (1:409,000–1:6,942,000)
	CPT II	1	1:2,466,615 (1:551,000–1:48,090,000
	CACTD	0	-
	LCHADD	17	1:145,000 (1:98,000–1:228,000)
			HADHA 1:145,000 (1:98,000–1:228,000)
			HADHB -
	MCADD	99	1:25,000 (1:21,200–1:29,700)
	MTPD	0	-
	PCD	11	1:225,000 (1:137,000–1:366,000)
	VLCADD	22	1:110,500 (1:1:72,100–1:176,000)
	GA II	12	Total 1:206,000 (1:128,400–1:329,000)
			ETFA 1:1,234,000 (1:408,130–1:3,726,900)
			ETFB 1:1,234,000 (1:408,130–1:3,726,900)
			ETFDH 1:309,000 (1:173,700–1:547,200)
			ETHE1 -
Organic acidemias	HMGA	1	1:2,466,615 (1:551,000–1:11,057,000)
	BKTD	4	1:617,000 (1:277,000–1:1,374,000)
	GA I	26	1:95,000 (1:69,000–1:130,000)
	IVA	17	1:145,000 (1:98,000–1:216,000)
	MMA-MCEE	0	-
	MMA-MUT	19	1:130,000 (1:90,000–1:189,000)
	cblA	5	1:494,000 (1:240,000–1:1,014,000)
	cblB	0	-
	cblC	2	1:1,234,000 (1:409,000–1:3,730,000)
	cblD	0	-
	PA	2	1:1,234,000 (1:409,000–1:3,730,000)
Urea cycle defects	ARG	2	1:1,234,000 (1:409,000–1:3,730,000)
	CIT I	17	1:145,000 (1:98,000–1:228,000)
	ASA	14	1:177,000 (1:114,000–1:292,000)
Other condition	HCSD	2	1:823,000 (1:329,000–1:2,057,000)
Total	IMDs with PKU	834	1:2900 (1:2770–1:3100)
	IMDs without PKU	296	1: 8170 (1:7400–1:9000)

The number of confirmed cases was defined as patients with an identified genotype in either a compound heterozygous or homozygous state. Cases of heterozygous carriers who had not yet undergone whole-genome sequencing were excluded from the disease frequency calculations. ARG = argininemia; ASA = argininosuccinic aciduria; BKTD = beta-ketothiolase deficiency; CACTD = carnitine-acylcarnitine translocase deficiency; cblA/B/C/D = cobalamin A/B/C/D defect; CH = congenital hypothyroidism; CIT I = citrullinemia type 1; CPT I/II = carnitine palmitoyltransferase I/II deficiency; FAOD = fatty acid β-oxidation disorders; GA I/II = glutaric acidemia type 1/2; HCSD = holocarboxylase synthetase deficiency; HCU = homocystinuria; HMGA = 3-hydroxy-3-methylglutaric aciduria; HPA = hyperphenylalaninemia; IVA = isovaleric acidemia; LCHAD = Long-chain 3-hydroxyacyl-CoA dehydrogenase deficiency; MCADD = medium chain acyl-CoA dehydrogenase deficiency; MMA-MCEE = methylmalonic acidemia due to methylmalonyl-CoA epimerase deficiency; MMA-MUT = methylmalonic aciduria due to methylmalonyl-coa mutase deficiency; MSUD = maple syrup urine disease; MTPD = mitochondrial trifunctional protein deficiency; PA = propionic acidemia; PCD = primary carnitine deficiency; PKU = phenylketonuria; TYR I = tyrosinemia type 1; VLCADD = very long chain acyl-CoA dehydrogenase deficiency.

**Table 3:** Frequency of common pathogenic variants identified during newborn screening in the Russian Federation.

Gene	Common Variant	Neonatal Screening (2023–2024) Allele, % (n)
*ACADM*	c.985A>G (p.Lys329Glu) c.388-19T>A (p.?)	38.38 (76) 10.10 (20)
*HADHA*	c.1528G>C (p.Glu510Gln)	79.41(27)
*GCDH*	c.1204C>T (p.Arg402Trp)	40.38 (21)
*FAH*	c.1025C>T (p.Pro342Leu)	30.76 (13)

**Table 4:** Summary of identified genetic variants and corresponding genes in patients with complex genotypes.

No.	Clinical Signs and Symptoms	Biochemical Tests	Identified Variants	
Patient 1 (39 weeks, 3350 g)	Asymptomatic at the time of examination	Primary screening: ↑C6, ↑C8, ↑C10, ↑C10:1, ↑Phe. Confirmatory testing: ↑Phe, ↑C8.	*ACADM* c.388-19T>A (p.?)/c.388-19T>A (p.?)	*PAH* c.1222C>T (p.Arg408Trp)/c.529G>A (p.Val177Met)
Patient 2 (38 weeks, 3580 g)	Asymptomatic at the time of examination	Primary screening: ↓C0 Confirmatory testing: ↓C0	*SLC22A5* c.641C>T (p.Ala214Val)/c.641C>T (p.Ala214Val)	*ETFB*:c.598A>G (p.Lys200Glu)/:c.598A>G (p.Lys200Glu)

↑ indicates an increase and ↓ indicates a decrease in the levels of acylcarnitines/amino acids.

**Table 5:** A comparison between SeIS (2021–2022) and NBS cohorts (2023–2024).

No	Disorder	Number of Confirmed Cases in 2021–2022 During the Selective Screening (SelS)	Number of Confirmed Cases in 2023–2024 During the Expanded NBS Program
1	MCADD	3	99
2	GA I	16	26
3	VLCADD	0	22
4	MMA-MUT (cblA, cblB, cblC, cblD, cblF, cblJ, cblX)	15	19
5	CIT I	5	17
6	IVA	1	17
7	LCHADD	5	17
8	TYR I	12	13

**Table 6:** PPV calculated for different groups of IMDs.

IEM	PPV, %
AA	24.8
OA (MS/MS)	0.6
FAOD	2.7
Total	3.87

## Data Availability

Original materials involved in the study are included in the article; additional requests may be sent to the corresponding author.

## Supplementary Materials

The following supporting information can be downloaded at https://www.mdpi.com/article/10.3390/ijns12010013/s1, Table S1: Cutoff values for amino acids and acylcarnitines, the elevation of which may indicate an inherited disorder included in the expanded NBS; Table S2: Cutoff values for organic acids, the elevation of which may indicate an inherited disorder included in the expanded NBS; Table S3: Hereditary disorders included in the NBS in the Russian Federation and the corresponding genes in the NGS panel; Table S4: PPV calculated for different IMDs.

## Abbreviations

The following abbreviations are used in this manuscript:

ARG	argininemia
ASA	argininosuccinic aciduria
BD	biotinidase deficiency
BH4	Tetrahydrobiopterin
BKTD	beta-ketothiolase deficiency
CACTD	carnitine-acylcarnitine translocase deficiency
CAH	congenital adrenal hyperplasia
CblA/B/C/D	cobalamin A/B/C/D defect
CIT1	citrullinemia type 1
CPT I/II	carnitine palmitoyltransferase I/II deficiency
DHPR deficiency	dihydropteridine reductase deficiency
FAOD	fatty acid β-oxidation disorders
GA I/II	glutaric acidemia type 1/2
GC-MS	gas chromatography-mass spectrometry
HCSD	holocarboxylase synthetase deficiency
HCU	homocystinuria
HMGA	3-hydroxy-3-methylglutaric aciduria
HPS	hyperphenylalaninemia
IMDs	inherited metabolic diseases
IVA	isovaleric acidemia
LCHAD	long-chain 3-hydroxyacyl-CoA dehydrogenase deficiency
MCADD	medium chain acyl-CoA dehydrogenase deficiency
MMA-MCEE	methylmalonic acidemia due to methylmalonyl-CoA epimerase deficiency
MMA-MUT	methylmalonic aciduria due to methylmalonyl-coa mutase deficiency
MS/MS	tandem mass-spectrometry
MSUD	maple syrup urine disease
MTP deficiency	mitochondrial trifunctional protein deficiency
NGS	next-generation sequencing
PCD	primary carnitine deficiency
PIDs	primary immunodeficiencies
PKU	phenylketonuria
SMA	spinal muscular atrophy
PTPS	6-pyruvoyl-tetrahydropterin synthase deficiency
TYR I	tyrosinemia type 1
VLCADD	very long chain acyl-CoA dehydrogenase deficiency
