# Supplementary material for: Two Years of Expanded Newborn Screening in Russia: High-Throughput Detection of Inherited Metabolic Disorders by Tandem Mass Spectrometry with Next-Generation Sequencing Confirmation
Source: Int J Neonatal Screen. 2026 Mar 2;12(1):13. doi: 10.3390/ijns12010013 (PMC13027380; doi:10.3390/ijns12010013)
Supplement: Supplementary file 1 [file IJNS-12-00013-s001.zip › IJNS-4100414-supplementary.pdf]

Supplementary Material

Table S1. Cutoff values for amino acids and acylcarnitines, the elevation of which may indicate an inherited disorder included in the expanded NBS.

№	Metabolites	Cut-off values (µmol/l)
1	AA Arg	> 70
2	AA Cit	> 80
3	AA Leu	> 380
4	AA Met	< 7 for HCU due to methylenetetrahydrofolate reductase deficiency
		> 80 for HCU due to Cystathionine Beta-Synthase Deficiency
5	AA Phe	> 120
6	AC C0	< 8 for Systemic carnitine deficiency
		> 90 for Carnitine palmitoyltransferase I deficiency
7	AC C14:1	> 0,32
8	AC C18OH	> 0,14
9	AC C3	> 5,5
10	AC C5	> 0,56
11	AC C5:1	> 0,15
12	AC C5DC	> 0,3
13	AC C5OH	> 0,5
14	AC C8	> 0,4
15	OA SuAc	> 2
16	Ratio C0/(C16+C18)	> 90
17	Ratio C3/C2	> 0,22

Table S2. Cutoff values for organic acids, the elevation of which may indicate an inherited disorder included in the expanded NBS.

№	Metabolites	Cut-off values (mmol/mol CRE)
1	2-hydroxyisovaleric acid	> 33.9
2	3-hydroxyglutaric acid	> 5.7
3	3-hydroxyisovaleric acid	> 83.0
4	3-hydroxypropionic acid	> 39.1
5	Glutaric acid	> 36.7
6	Methylmalonic acid	> 67.8
7	Orotic acid	> 26.6
8	Ethylmalonic acid	> 56.7
9	3-methylcrotonylglycine	> 4.9
10	Isovalerylglycine	> 16.5
11	Propionylglycine	> 2.6
12	Tiglylglycine	> 17.9

Table S3. Hereditary disorders included in the NBS in the Russian Federation and the corresponding genes in the NGS panel.

	N	Disorder (OMIM)	Genes	Ref seq
Aminoacidopathies	1	Homocystinuria (236200)	*CBS*	NM_000071.3
	2 *	Homocystinuria without methylmalonic aciduria, methylcobalamin deficiency type cblE (236270)	*MTRR*	NM_002454.3
	3 *	Homocystinuria without methylmalonic aciduria, methylcobalamin deficiency type cblG (250940)	*MTR*	NM_000254.3
	4 *	Homocystinuria due to methylenetetrahydrofolate reductase deficiency (236250)	*MTHFR*	NM_005957.5
	5	Maple syrup urine disease (248600, 620698, 620699, 246900, 615135)	*BCKDHA*	NM_000709
	6		*BCKDHB*	NM_183050.4
	7		*DBT*	NM_001918.5
	8		*DLD*	NM_000108.5
	9 *		*PPM1K*	NM_152542.5
	10	Tyrosinemia type 1 (276700)	*FAH*	NM_000137.4
	11 *	Tyrosinemia type 2 (276600)	*TAT*	NM_000353.3
	12	Phenylketonuria (261600)	*PAH*	NM_000277.3
	13	Hyperphenylalaninemia, BH4-deficient, A (261640)	*PTS*	NM_000317.3
	14	Hyperphenylalaninemia, BH4-deficient, B (233910)	*GCH1*	NM_000161.3
	15	Hyperphenylalaninemia, BH4-deficient, C (261630)	*QDPR*	NM_000320.3
	16	Hyperphenylalaninemia, BH4-deficient, D (264070)	*PCBD1*	NM_000281.4
	17 *	Dystonia, dopa-responsive, due to sepiapterin reductase deficiency (612716)	*SPR*	NM_003124.5
	18	Hyperphenylalaninemia, mild, non-BH4-deficient (617384)	*DNAJC12*	NM_021800.3
	19	Nonketotic hyperglycinemia (620398, 605899, 620423)	*AMT*	NM_000481
	20		*GCSH*	NM_004483.5
	21		*GLDC*	NM_000170.3
Fatty acid oxidation disorders (FAOD)	22	Medium chain acyl-CoA dehydrogenase deficiency (201450)	*ACADM*	NM_000016.6
	23 *	Short-chain acyl-CoA dehydrogenase deficiency (201470)	*ACADS*	NM_000017.4
	24	Very long-chain acyl-CoA dehydrogenase deficiency (201475)	*ACADVL*	NM_000018.4
	25	Long-chain 3-hydroxyacyl-CoA dehydrogenase (609016)	*HADHA*	NM_000182.5
	26		*HADHB*	NM_000183.3
	27 *	3-alpha-hydroxyacyl-CoA dehydrogenase deficiency	*HADH*	NM_005327.7
	28	Carnitine palmitoyltransferase I deficiency	*CPT1A*	NM_001876.4
	29		*CPT1B*	NM_152246.3
	30	Carnitine palmitoyltransferase II deficiency	*CPT2*	NM_000098.3
	31 *	Carnitine-acylcarnitine translocase deficiency (613698)	*SLC25A20*	NM_000387.6
	32	Systemic carnitine deficiency (603377)	*SLC22A5*	NM_003060.4
Organic acidemias	33	3-hydroxy-3 methylglutaric aciduria (246450)	*HMGCL*	NM_000191.3
	34 *	Combined malonic and methylmalonic acidemia (614265)	*ACSF3*	NM_174917.5
	35	Beta-ketothiolase deficiency (203750)	*ACAT1*	NM_000019

	36	Glutaric acidemia type 1 (231670)	*GCDH*	NM_000159.4
	37	Glutaric acidemia type 2 (231680)	*ETFA*	NM_000126.4
	38		*ETFB*	NM_001985.3
	39		*ETFDH*	NM_004453.4
	40 *	Glutaric acidemia type 3 (231690)	*SUGCT*	NM_024728.3
	41	Isovaleric acidemia (243500)	*IVD*	NM_002225.5
	42 *	Malonic acid (Malonyl-CoA decarboxylase deficiency) (248360)	*MLYCD*	NM_012213.3
	43 *	Combined immunodeficiency-megaloblastic anemia due to methylenetetrahydrofolate dehydrogenase 1 deficiency (617780)	*MTHFD1*	NM_005956.4
	44 *	Transcobalamin deficiency (275350)	*TCN2*	NM_000355.4
	45	Methylmalonic acidemia with homocystinuria, type cblA (277400)	*MMAA*	NM_172250.3
	46	Methylmalonic acidemia with homocystinuria, type cblB (251110)	*MMAB*	NM_052845.4
	47	Methylmalonic aciduria due to methylmalonyl-coa mutase deficiency (251000)	*MMUT*	NM_000255.4
	48	Methylmalonic acidemia with homocystinuria, type cblC (277400)	*MMACHC*	NM_015506.3
	49	Methylmalonic acidemia with homocystinuria, type cblD (277410)	*MMADHC*	NM_015702.3
	50	Methylmalonyl-CoA epimerase deficiency (608419)	*MCEE*	NM_032601.4
	51 *	Mitochondrial DNA depletion syndrome 5 (encephalomyopathic with or without methylmalonic aciduria) (612073)	*SUCLA2*	NM_003850.3
	52 *	Mitochondrial DNA depletion syndrome (615418)	*SUCLG1*	NM_003849.4
	53 *		*SUCLG2*	NM_001177599.2
	54 *	Methylmalonic aciduria and homocystinuria, cblC type, digenic (277400)	*PRDX1*	NM_002574.4
	55 *	Methylmalonic acidemia with homocystinuria, type cblF (277380)	*LMBRD1*	NM_018368.4
	56 *	Methylmalonic acidemia with homocystinuria, type cblJ (614857)	*ABCD4*	NM_005050
	57 *	Methylmalonic acidemia with homocystinuria, type cblX (309541)	*HCFC1*	NM_005334.3
	58	Propionic acidemia (606054)	*PCCA*	NM_000282.4
	59		*PCCB*	NM_000532.5
	60 *	Methylmalonic aciduria due to transcobalamin receptor defect (613646)	*CD320*	NM_016579.4
	61 *	3-methylcrotonyl-CoA carboxylase deficiency (210200, 210210)	*MCCC1*	NM_020166.5
	62 *		*MCCC2*	NM_022132.5
	63 *	D-2-hydroxyglutaric aciduria 1 (600721)	*D2HGDH*	NM_152783.5
	64 *	D-2-hydroxyglutaric aciduria 2 (613657)	*IDH2*	NM_002168.4
	65 *	L-2-hydroxyglutaric aciduria (236792)	*L2HGDH*	NM_024884.3
	66 *	Combined D-2- and L-2-hydroxyglutaric aciduria (615182)	*SLC25A1*	NM_005984.5
	67 *	Ethylmalonic encephalopathy (602473)	*ETHE1*	NM_014297.5
Urea cycle defects	68 *	Argininemia (207800)	*ARG1*	NM_000045
	69 *	Argininosuccinic aciduria (207900)	*ASL*	NM_000048
	70	Citrullinemia type 1 (215700)	*ASS1*	NM_000050.4
	71 *	Citrullinemia type 2 (603471)	*SLC25A1*	NM_005984.5
	72 *	Carbamoyl-phosphate synthetase 1 deficiency (237300)	*CPS1*	NM_001875.5
	73 *	Ornithine transcarbamylase deficiency (311250)	*OTC*	NM_000531.6

	74 *	Hyperammonemia due to N-acetylglutamate synthase deficiency (237310)	*NAGS*	NM_153006.3
	75 *	Hyperornithinemia-hyperammonemia-homocitrullinuria syndrome (238970)	*SLC25A15*	NM_014252.4
	76 *	Gyrate atrophy of choroid and retina with or without ornithinemia (258870)	*OAT*	NM_000274.4
	77 *	Hyperammonemic encephalopathy due to carbonic anhydrase VA deficiency (615751) *	*CA5A*	NM_001739.2
Other conditions	78	Biotinidase deficiency (253260)	*BTD*	NM_001370658.1
	79	Holocarboxylase synthetase deficiency (253270)	*HLCS*	NM_000411.8
	80 *	Lipid storage myopathy due to flavin adenine dinucleotide synthetase deficiency (255100)	*FLAD1*	NM_025207.5
	81 *	Brown-Vialetto-Van Laere syndrome 1 (211530)<br>Fazio-Londe disease (211500)	*SLC52A3*	NM_033409.4
	82 *	Brown-Vialetto-Van Laere syndrome 2 (614707)	*SLC52A2*	NM_024531.5
	83 *	Riboflavin deficiency (615026)	*SLC52A1*	NM_017986.4
	84 *	Exercise intolerance, riboflavin-responsive (616839)	*SLC25A32*	NM_030780.5
	85 *	X-linked disorder adrenoleukodystrophy (300371)	*ABCD1*	NM_000033.4
	86 *	Adenosine kinase deficiency (614300)	*ADK*	NM_006721.4
	87 *	Primary hyperoxaluria type I (259900)	*AGXT*	NM_000030.3
	88 *	Hypermethioninemia with S-adenosylhomocysteine hydrolase deficiency (613752)	*AHCY*	NM_000687.4
	89 *	Aromatic L-amino acid decarboxylase deficiency (608643)	*DDC*	NM_001082971.2
	90 *	Deoxyguanosine kinase deficiency (601465)	*DGUOK*	NM_080916.3
	91 *	Glycine N-methyltransferase deficiency (606628)	*GNMT*	NM_018960.6
	92 *	Methionine adenosyltransferase deficiency (610550)	*MAT1A*	NM_000429.3
*** - Other conditions included in NGS panel but not included in the expanded NBS program**<br>**The conditions highlighted in orange are not validated at the Research Centre for Medical Genetics in Moscow (the national reference centre).**				

Table S4. PPV calculated for different IMDs.

**IMD**	**Samples delivered for confirmatory diagnosis**	**Number of confirmed cases in 2023-2024**	**PPV, %**
PKU/HPA	1220	538	44,1
HCU	128	4	3,1
MSUD	354	4	1,1
TYR I	21	13	61,9
ARG	312	2	0,6
CIT I	212	17	8,0
ASA	140	14	10,0

CPT I	3	2	66,7
CPT II	133	1	0,8
LCHADD	17	17	100,0
MCADD	339	99	29,2
PCD	1276	11	0,9
VLCADD	4291	22	0,5
GA II	52	12	23,1
BKTD	15	4	26,7
GA I	86	26	30,2
IVA	1832	17	0,9
MMA & PA	7918	28	0,4
HCSD	3176	2	0,1
